# Supplementary material for: Cleavage of CAD by caspase-3 determines the cancer cell fate during chemotherapy
Source: Nat Commun. 2025 May 30;16:5006. doi: 10.1038/s41467-025-60144-2 (PMC12123037; doi:10.1038/s41467-025-60144-2)
Supplement: Supplementary file 1 — Supplementary Information [file 41467_2025_60144_MOESM1_ESM.pdf]

Supplementary Information

Cleavage of CAD by caspase-3 determines the cancer cell fate during chemotherapy

Jingsong Ma, Jiabao Zhao, Chensong Zhang, Jinshui Tan, Ao Cheng, Zhuo Niu, Zeyang Lin, Guangchao Pan, Chao Chen, Yang Ding, Mengya Zhong, Yifan Zhuang, Yubo Xiong, Huiwen Zhou, Shengyi Zhou, Meijuan Xu, Wenjie Ye, Funan Li, Yongxi Song, Zhenning Wang, Xuehui Hong

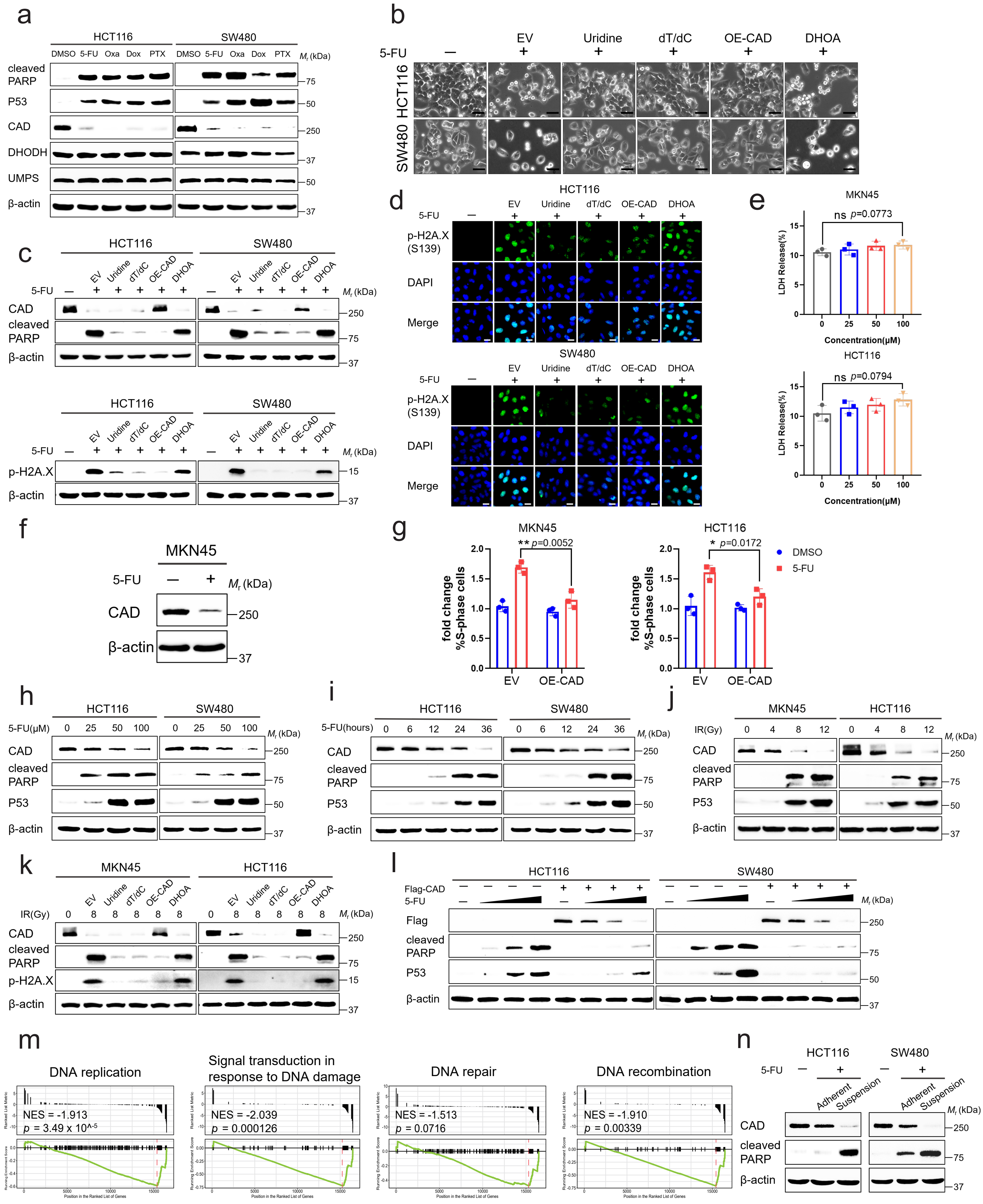
a
HCT116
SW480
DMSO
5-FU
Oxa
Dox
PTX
Mr (kDa)
cleaved PARP
P53
CAD
DHODH
UMPS
β-actin
75
50
250
37
b
EV
Uridine
dT/dC
OE-CAD
DHOA
5-FU
HCT116
SW480
c
d
p-H2A.X (S139)
DAPI
Merge
e
MKN45
ns p=0.0773
ns p=0.0794
LDH Release(%)
Concentration(μM)
f
g
** p=0.0052
* p=0.0172
fold change %S-phase cells
DMSO
h
5-FU(μM)
i
5-FU(hours)
j
IR(Gy)
k
l
Flag-CAD
Flag
m
DNA replication
NES = -1.913
p = 3.49 x 10^-5
Signal transduction in response to DNA damage
NES = -2.039
p = 0.000126
DNA repair
NES = -1.513
p = 0.0716
DNA recombination
NES = -1.910
p = 0.00339
n
Adherent
Suspension

Supplementary Fig.1 CAD levels show a downward trend under chemotherapy.

(**a**) Western blot showing cleaved PARP, P53, CAD, DHODH and UMPS protein levels in HCT116 and SW480 cells were treated with 5-FU (50 μM), Oxa (20 μM), Dox (20 μM) and PTX (10 nM) for 24 h.

(**b**) Representative photomicrographs of HCT116 and SW480 cells expressing empty vector (EV), CAD, or supplemented with Uridine (100 μM), dT/dC (20 μM) and DHOA (250 μM), then treated with 5-FU (50 μM). Scale bar, 50 μm.

(**c-d**) Representative Immunoblot analysis (c) with CAD, cleaved PARP, and p-H2A.X antibody and Immunofluorescence (d) with p-H2A.X antibody of HCT116 and SW480 cells as described in (b). Scale bar, 20 μm.

(**e**) Culture supernatants of 5-FU treated MKN45 and HCT116 cells with a concentration gradient were collected to measure the percentage of LDH release. n = 3 biologically independent samples per group.

(**f**) Immunoblot analysis of MKN45 cells treated with 5-FU.

(**g**) Propidium Iodide cell-cycle analysis of MKN45 and HCT116 cells transfected with Flag-CAD or EV and treated with 5-FU or DMSO. n = 3 biologically independent samples per group.

(**h-i**) Protein expression of CAD, cleaved PARP and P53 were determined by Western blot after HCT116 and SW480 cells were treated with different concentrations (0, 25, 50, and 100 μM) and time period (0, 6, 12, 24, and 36 h) of 5-FU.

(**j**) Protein expression of CAD, cleaved PARP and P53 were determined by Western blot after MKN45 and HCT116 cells were treated with different doses of ionizing

radiation (0, 4, 8 and 12 Gy).

(**k**) Representative Immunoblot analysis with Flag, cleaved PARP, and p-H2A.X antibody of MKN45 and HCT116 cells expressing EV, CAD, or supplemented with Uridine (100 μM), dT/dC (20 μM) and DHOA (250 μM), then treated with ionizing radiation (8 Gy).

(**l**) HCT116 and SW480 cells overexpressing Flag-CAD were treated with different concentrations of 5-FU for 24h. Flag, cleaved PARP, P53 and β-actin expression were detected.

(**m**) Gene set enrichment analysis of DNA replication, signal transduction in response to DNA damage, DNA repair and DNA recombination pathways in MKN45-EV cells treated with 5-FU (50 μM) versus MKN45-CAD cells treated with 5-FU (50 μM).

(**n**) Western blot analysis of CAD, cleaved PARP protein expression in 5-FU (100 μM) treatment still attached and suspended cells.

Data in panels **a**, **c**, **h**, **i**, **j**, **k** and **n** derive from the same biological experiment but were processed on parallel gels (see Source Data for gel-specific details).

Data were expressed as means±SD, two-tailed Student's *t* test. ns: not significant, $^*p < 0.05$, $^{**}p < 0.01$.

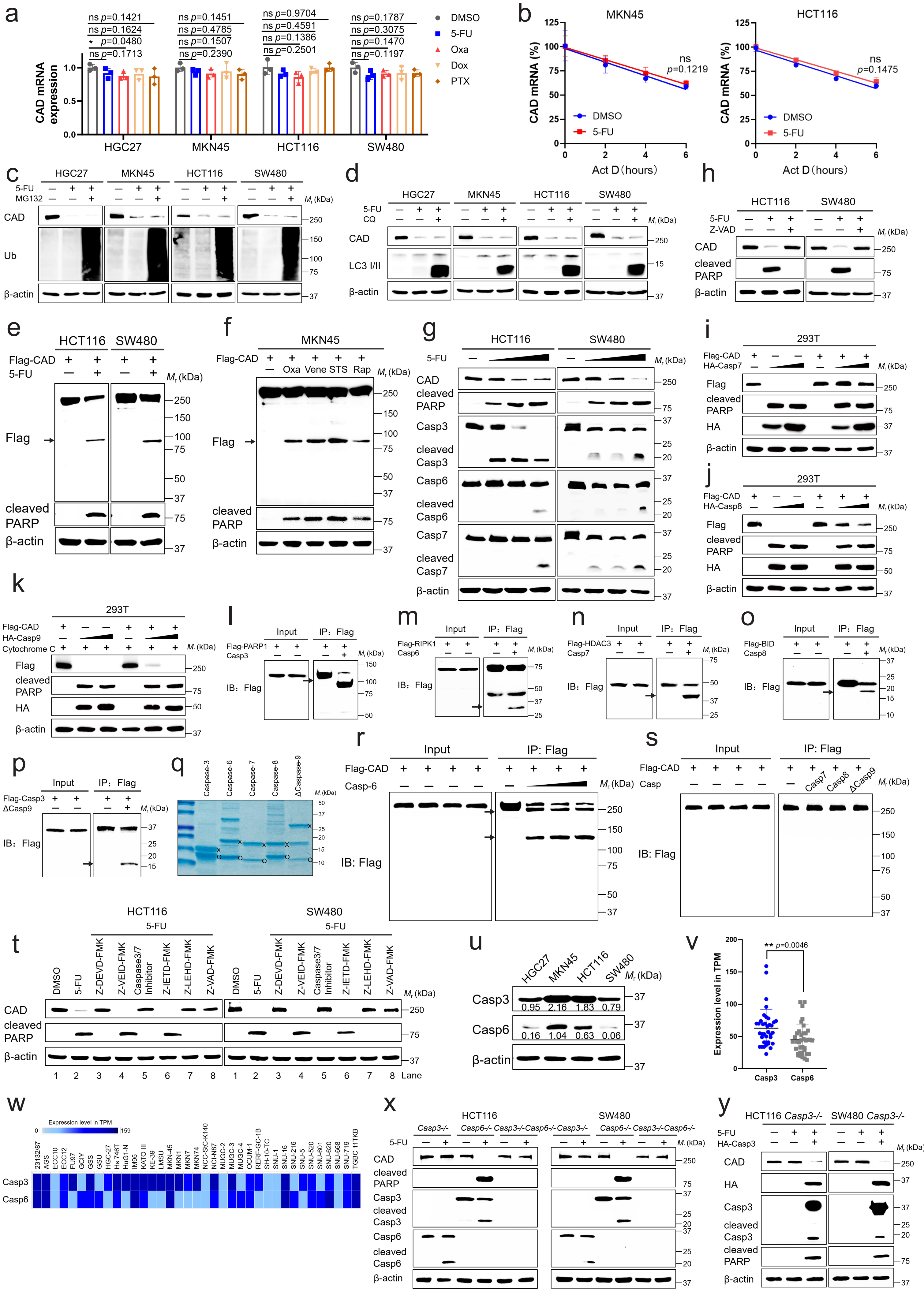

a
ns p=0.1421
ns p=0.1624
* p=0.0480
ns p=0.1713
ns p=0.1451
ns p=0.4785
ns p=0.1507
ns p=0.2390
ns p=0.9704
ns p=0.4591
ns p=0.1386
ns p=0.2501
ns p=0.1787
ns p=0.3075
ns p=0.1470
ns p=0.1197
CAD mRNA expression
DMSO
5-FU
Oxa
Dox
PTX
HGC27
MKN45
HCT116
SW480
b
MKN45
HCT116
CAD mRNA (%)
ns p=0.1219
ns p=0.1475
DMSO
5-FU
Act D (hours)
c
5-FU
MG132
CAD
Ub
β-actin
Mr (kDa)
d
CQ
LC3 I/II
h
Z-VAD
cleaved PARP
e
Flag-CAD
Flag
f
Oxa
Vene
STS
Rap
g
Casp3
cleaved Casp3
Casp6
cleaved Casp6
Casp7
cleaved Casp7
i
293T
HA-Casp7
HA
j
HA-Casp8
k
HA-Casp9
Cytochrome C
l
Input
IP: Flag
Flag-PARP1
IB: Flag
m
Flag-RIPK1
n
Flag-HDAC3
o
Flag-BID
p
Flag-Casp3
ΔCasp9
q
Caspase-3
Caspase-6
Caspase-7
Caspase-8
ΔCaspase-9
r
Casp-6
s
Casp
Casp7
Casp8
ΔCasp9
t
DMSO
Z-DEVD-FMK
Z-VEID-FMK
Caspase3/7 Inhibitor
Z-IETD-FMK
Z-LEHD-FMK
Z-VAD-FMK
Lane
u
0.95 2.16 1.83 0.79
0.16 1.04 0.63 0.06
v
** p=0.0046
Expression level in TPM
Casp3
Casp6
w
Expression level in TPM
23132/87 AGS ECC10 ECC12 FU97 GCIY GSS GSU HGC-27 Hs 746T HuG1-N IM95 KATO III KE-39 LMSU MKN-45 MKN1 MKN7 MKN74 NCC-StC-K140 NCI-N87 MUGC-2 MUGC-3 MUGC-4 OCUM-1 RERF-GC-1B SH-10-TC SNU-1 SNU-16 SNU-216 SNU-5 SNU-520 SNU-601 SNU-620 SNU-668 SNU-719 TGBC 11TKB
x
Casp3-/-
Casp6-/-
Casp3-/-Casp6-/-
y
HCT116 Casp3-/-
SW480 Casp3-/-
HA-Casp3

Supplementary Fig.2 CAD as a caspase-3 cleavage substrate.

(**a**) qRT-PCR showing the relative expression of CAD mRNA in HGC27, MKN45, HCT116, and SW480 cell lines were treated with 5-FU (50 μM), Oxa (20 μM), Dox (20 μM) and PTX (10 nM) for 24h. n = 3 biologically independent samples per group.

(**b**) CAD mRNA stability was determined using actinomycin D (ActD) mRNA stability assays in MKN45 and HCT116 cells. n = 3 biologically independent samples per group.

(**c-d**) HGC27, MKN45, HCT116, and SW480 cells were treated with 5-FU (50 μM) in the absence and presence of MG-132 (5 μM) or Chloroquine (20 μM) for 24 h. Total cell lysates were immunoblotted with anti-CAD, Ub, LC3I/II, or β-actin antibody.

(**e**) Western blot of HCT116 and SW480 cells transfected with the indicated CAD construct and treated for 24 h with 5-FU.

(**f**) Western blot of MKN45 cells transfected with the indicated CAD construct and treated for 24 h with Oxa (20 μM), Vene (10 μM), STS (1 μM), and Rap (5 μM).

(**g**) Immunoblotting analysis of the indicated proteins in HCT116 and SW480 cells were treated with 5-FU with concentration gradients. The 5-FU-treated groups show cleavage of PARP and caspase-3/6/7 with a successive decline in CAD expression levels.

(**h**) HCT116 and SW480 cells were treated with 5-FU (50 μM) in the absence and presence of Z-VAD (20 μM) for 24 h. Total cell lysates were immunoblotted with anti-CAD, cleaved PARP, or β-actin antibody.

(**i-k**) HEK-293T cells were co-transfected with Flag-CAD and HA-caspase-7 (i) or HA-caspase-8 (j). Flag, cleaved PARP, HA and β-actin expression were detected by

immunoblotting. The same indicators were detected in the whole cell lysates co-transfected with HA-caspase-9 and additional supplementation of cytochrome C (k).

(**l-p**) Western blot of in vitro cleavage reaction containing purified Flag-PARP1 (l), Flag-RIPK1 (m), Flag-HDAC3 (n), Flag-BID (o) and Flag-caspase-3 (p) by immunoprecipitation and recombinant active human caspase-3/6/7/8/△9 correspondingly.

(**q**) SDS–PAGE (15%) analysis of purified samples of caspase-3/6/7/8/△9. The large (x) and small (o) subunits of each caspase appear as predominant bands.

(**r**) Western blot of in vitro cleavage reaction containing purified Flag-CAD by immunoprecipitation and fourfold serial dilutions of recombinant active caspase-6, starting from a lowest concentration of 0 ng/μl.

(**s**) Western blot of in vitro cleavage reaction containing purified Flag-CAD by immunoprecipitation and recombinant active human caspase- caspase-7/8/△9.

(**t**) HCT116 and SW480 cells were treated with 5-FU in the presence of various indicated caspase inhibitors (the final concentration of all caspase inhibitors was 20 μM except for caspase-3/7 Inhibitor, whose concentration was 200 nM). 24 h after drug treatment, cells were collected and lysates were subjected to immunoblot using CAD, cleaved PARP and β-actin antibody.

(**u**) Protein expression of caspase-3 and caspase-6 were determined by Western blot in HGC27, MKN45, HCT116 and SW480 cell lines.

(**v-w**) Through gene query in Expression Atlas database and screening search results according to different conditions, the gene expression differences of caspase-3 and

caspase-6 in all human GC cell lines were compared and paired analysis was carried out simultaneously.

(**x**) *casp3-/-*, *casp6-/-* and *casp3-/-casp6-/-* HCT116 and SW480 cells were stimulated for 24 h with 5-FU, followed by immunoblot analysis.

(**y**) *casp3-/-* HCT116 and SW480 cells were treated with 5-FU (50 μM) in the presence or absence of recovery of HA-caspase-3. The levels of indicated proteins were determined by immunoblotting.

Data in panels **c**, **d**, **e**, **f**, **g**, **h**, **l**, **m**, **p**, **r**, **s**, **u**, **x** and **y** derive from the same biological experiment but were processed on parallel gels (see Source Data for gel-specific details). The cleavage product is highlighted by arrowheads. Data were expressed as means±SD, two-tailed Student's *t* test. ns: not significant, *p <0.05, **p <0.01.

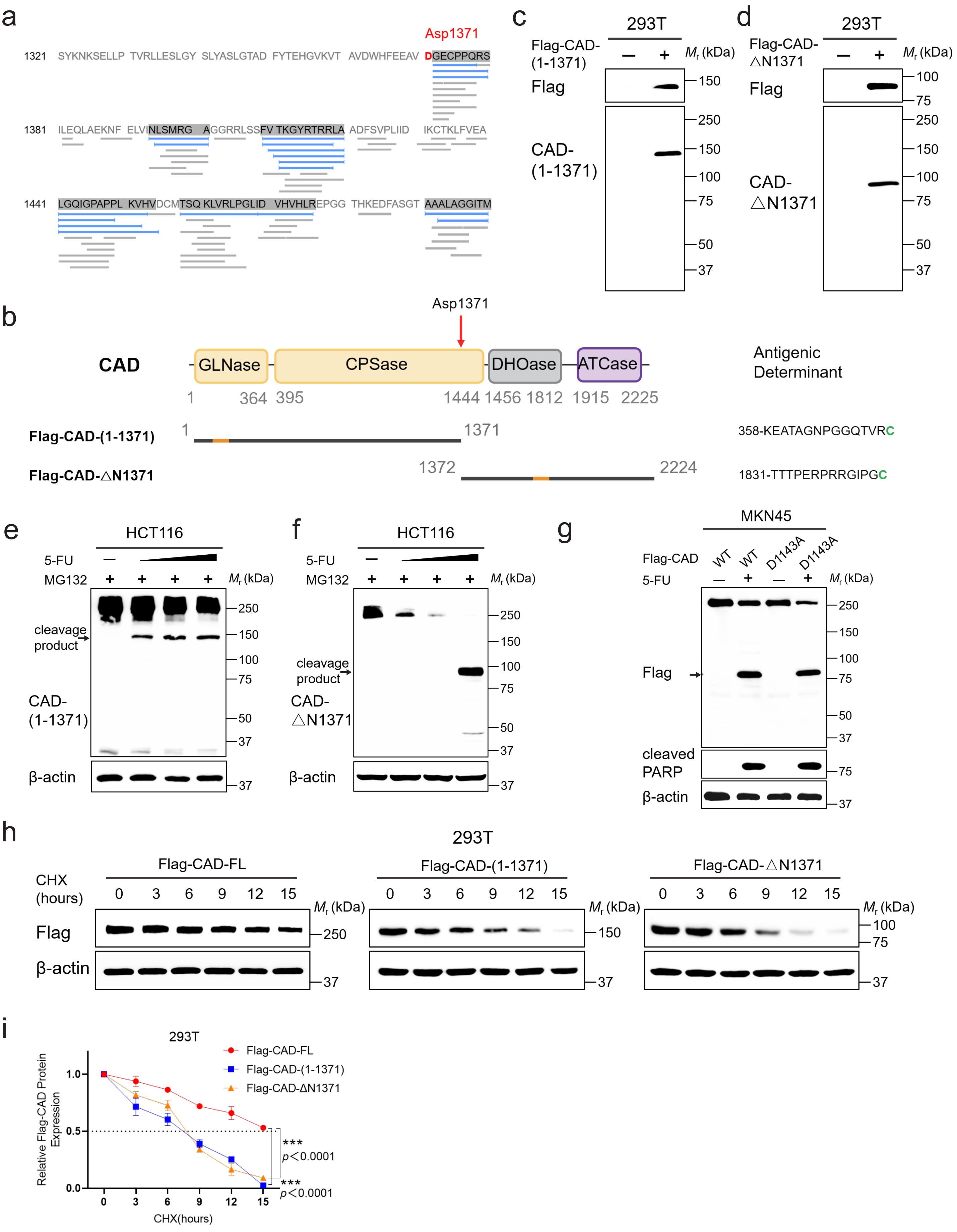
a
Asp1371
1321 SYKNKSELLP TVRLLESLGY SLYASLGTAD FYTEHGVKVT AVDWHFEEAV DGECPPQRS
1381 ILEQLAEKNF ELVINLSMRG AGGRRLSSFV TKGYRTRRLA ADFSVPLIID IKCTKLFVEA
1441 LGQIGPAPPL KVHVDCMTSQ KLVRLPGLID VHVHLREPGG THKEDFASGT AAALAGGITM
b
Asp1371
CAD
GLNase
CPSase
DHOase
ATCase
1 364 395 1444 1456 1812 1915 2225
Antigenic Determinant
Flag-CAD-(1-1371)
Flag-CAD-△N1371
358-KEATAGNPGGQTVRC
1831-TTTPERPRRGIPGC
c
293T
Flag-CAD-(1-1371)
Flag
CAD-(1-1371)
d
293T
Flag-CAD-△N1371
Flag
CAD-△N1371
e
HCT116
5-FU
MG132
cleavage product
CAD-(1-1371)
β-actin
f
HCT116
5-FU
MG132
cleavage product
CAD-△N1371
β-actin
g
MKN45
Flag-CAD WT WT D1143A D1143A
5-FU
Flag
cleaved PARP
β-actin
h
293T
Flag-CAD-FL
Flag-CAD-(1-1371)
Flag-CAD-△N1371
CHX (hours)
0 3 6 9 12 15
Flag
β-actin
M_r (kDa)
i
293T
Flag-CAD-FL
Flag-CAD-(1-1371)
Flag-CAD-ΔN1371
Relative Flag-CAD Protein Expression
CHX(hours)
*** p<0.0001
*** p<0.0001

Supplementary Fig.3 Caspase-3 cleaves CAD at the Asp1371 site.

(**a**) Shotgun proteomic analysis based on Parallel Accumulation Serial Fragmentation (PASEF), showing accurate sequence of C-terminal fragment protein of CAD after caspase-3 cleavage.

(**b**) Schematic representation of CAD being cleaved at Aspartic acid 1371. Polypeptide sequences represent antigenic determinants that recognize N-and C-terminal fragments.

(**c-d**) HEK-293T cells were overexpressed Flag-CAD-(1-1371) and Flag-CAD-△N1371 respectively. The cell lysates were then subjected to immunoblotting analyses using anti-CAD-(1-1371) (c) and anti-CAD-△N1371 (d) antibodies. Anti-Flag antibody was used to ensure the expression of the target protein.

(**e-f**) HCT116 cells were co-treated MG-132 and concentration gradient of 5-FU for 24 h. Cell lysates were subjected to immunoblotting using anti-CAD-(1-1371) (e) and anti-CAD-△N1371 antibodies (f).

(**g**) Western blot of MKN45 cells transfected with the indicated CAD constructs and treated for 24 h with 5-FU.

(**h**) HEK-293T cells were transfected with Flag-CAD-FL, Flag-CAD-(1-1371) and Flag-CAD-△N1371 and CHX (20 μg/ml) was added to cells 24 h after transfection. Cells were collected after indicated time (0, 3, 6, 9, 12, or 15 h) and cell lysates were immunoblotted with anti-Flag antibody to detect the protein level change and with anti-β-actin antibody as a loading control.

(**i**) Scanning densitometry was performed for each Western blot indicated in (h) using ImageJ. The best-fit exponential decay lines were plotted using GraphPad Prism and

the half-life can be read from it (marked by dot-dash line). n = 3 biologically independent samples per group.

Data in panels **c**, **d**, **e**, **f** and **g** derive from the same biological experiment but were processed on parallel gels (see Source Data for gel-specific details).

Arrowhead highlights band corresponding to cleavage. Data were expressed as means±SD, two-tailed Student's *t* test. ***p <0.001.

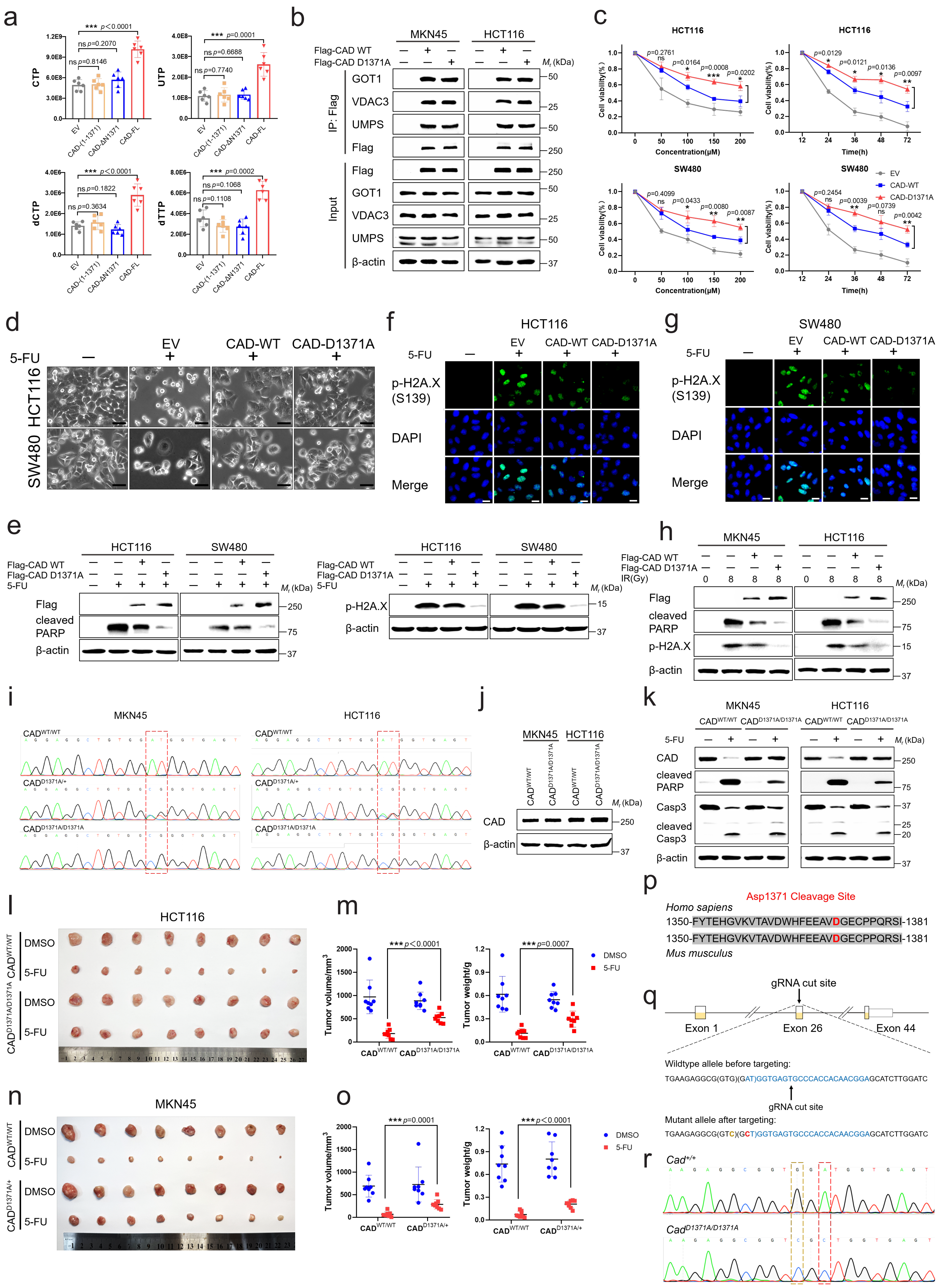

a
CTP
UTP
dCTP
dTTP
EV
CAD-(1-1371)
CAD-ΔN1371
CAD-FL
b
MKN45
HCT116
Flag-CAD WT
Flag-CAD D1371A
IP: Flag
Input
GOT1
VDAC3
UMPS
Flag
β-actin
c
HCT116
SW480
Cell viability(%)
Concentration(μM)
Time(h)
EV
CAD-WT
CAD-D1371A
d
5-FU
EV
CAD-WT
CAD-D1371A
HCT116
SW480
e
Flag
cleaved PARP
p-H2A.X
β-actin
f
g
p-H2A.X (S139)
DAPI
Merge
h
IR(Gy)
i
MKN45
HCT116
j
CAD
k
Casp3
cleaved Casp3
l
DMSO
5-FU
m
Tumor volume/mm3
Tumor weight/g
n
o
p
Asp1371 Cleavage Site
Homo sapiens
1350-FYTEHGVKVTAVDWHFEEAVDGECPPQRSI-1381
1350-FYTEHGVKVTAVDWHFEEAVDGECPPQRSI-1381
Mus musculus
q
gRNA cut site
Exon 1
Exon 26
Exon 44
Wildtype allele before targeting:
TGAAGAGGCG(GTG)(GAT)GGTGAGTGCCCACCACAACGGAGCATCTTGGATC
Mutant allele after targeting:
TGAAGAGGCG(GTC)(GCT)GGTGAGTGCCCACCACAACGGAGCATCTTGGATC
r

Supplementary Fig.4 CAD cleavage plays a critical role in mediating the effects of chemotherapy.

(**a**) Relative quantification of CTP, UTP, dCTP and dTTP levels in MKN45-EV, MKN45-CAD-(1-1371), MKN45-CAD-△N1371 and MKN45-CAD-FL (full-length) cells. n = 6 biologically independent samples per group.

(**b**) Immunoprecipitation (IP) analysis of the interaction between Flag-CAD WT or Flag-CAD D1371A with endogenous GOT1, VDAC3, and UMPS in MKN45 and HCT116 cells with anti-Flag antibody.

(**c**) Cell death assays of HCT116-EV, HCT116-CAD-WT or HCT116-CAD-D1371A cells treated with different concentrations and time period of 5-FU as indicated. Repeat the same treatment and experimental design in SW480 cells. n = 3 biologically independent samples per group.

(**d**) Representative photomicrographs of HCT116 and SW480 cells expressing EV, CAD-WT, or CAD-D1371A, then treated with 5-FU. Scale bar, 50 μm.

(**e-g**) Representative Immunoblot analysis (e) with Flag, cleaved PARP, and p-H2A.X antibody and Immunofluorescence (f-g) with p-H2A.X antibody of HCT116 and SW480 cells as described in (d). Scale bar, 20 μm.

(**h**) Representative Immunoblot analysis with Flag, cleaved PARP, and p-H2A.X antibody of MKN45 and HCT116 cells expressing EV, CAD-WT, or CAD-D1371A, then treated with ionizing radiation (8 Gy).

(**i**) The sequencing validation of D1371A of CAD in cell lines.

(**j**) Immunoblotting analysis of CAD expression in $CAD^{WT/WT}$ or homozygous

$CAD^{D1371A/D1371A}$ cells.

(**k**) Immunoblotting analysis of CAD, cleaved PARP, and Casp3 expression in 5-FU-treated $CAD^{WT/WT}$ or homozygous $CAD^{D1371A/D1371A}$ cells.

(**l-m**) Size of tumors formed by injected subcutaneously with $CAD^{WT/WT}$ or homozygous $CAD^{D1371A/D1371A}$ HCT116 cells in xenograft mouse models were treated with DMSO and 5-FU separately and statistical analysis. n = 8 biologically independent samples per group.

(**n-o**) Size of tumors formed by injected subcutaneously with $CAD^{WT/WT}$ or heterozygous $CAD^{D1371A//+}$ MKN45 cells in xenograft mouse models were treated with DMSO and 5-FU separately and statistical analysis. n = 8 biologically independent samples per group.

(**p**) Sequence alignment of human and mouse CAD at indicated amino acid residues. The recognition site is marked by red highlight.

(**q**) A schematic diagram of the murine *Cad* locus and the *Cad*-D1371A allele.

(**r**) Tail genotyping for the $Cad^{D1371A/D1371A}$ mutant mice by sequencing.

Data in panels **b**, **e**, **h** and **k** derive from the same biological experiment but were processed on parallel gels (see Source Data for gel-specific details).

Data were expressed as means±SD, two-tailed Student's *t* test. ns: not significant, *p <0.05, **p <0.01, ***p <0.001.

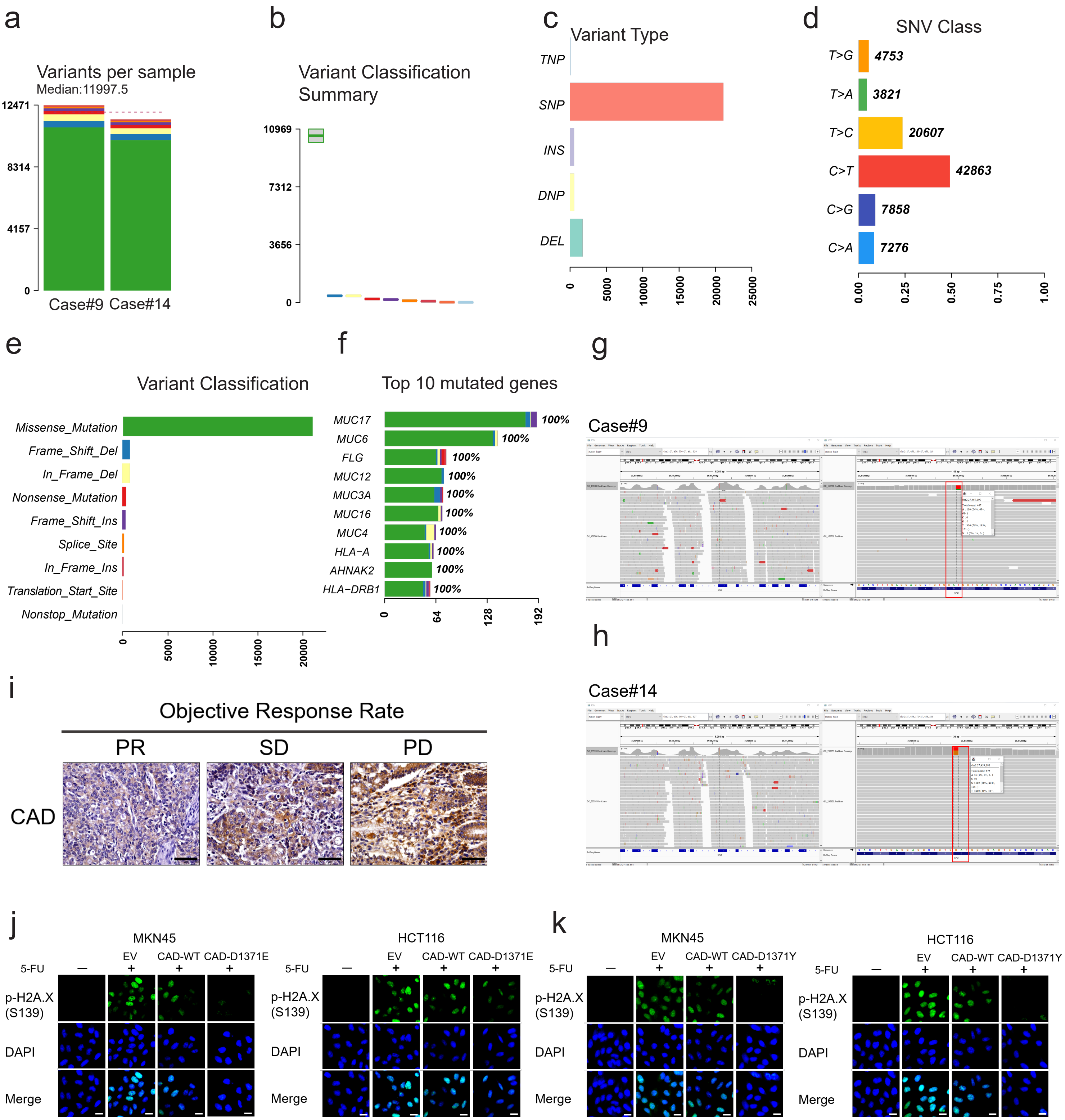
a
Variants per sample
Median:11997.5
12471
8314
4157
0
Case#9 Case#14
b
Variant Classification Summary
10969
7312
3656
0
c
Variant Type
TNP
SNP
INS
DNP
DEL
0 5000 10000 15000 20000 25000
d
SNV Class
T>G 4753
T>A 3821
T>C 20607
C>T 42863
C>G 7858
C>A 7276
0.00 0.25 0.50 0.75 1.00
e
Variant Classification
Missense_Mutation
Frame_Shift_Del
In_Frame_Del
Nonsense_Mutation
Frame_Shift_Ins
Splice_Site
In_Frame_Ins
Translation_Start_Site
Nonstop_Mutation
0 5000 10000 15000 20000
f
Top 10 mutated genes
MUC17 100%
MUC6 100%
FLG 100%
MUC12 100%
MUC3A 100%
MUC16 100%
MUC4 100%
HLA−A 100%
AHNAK2 100%
HLA−DRB1 100%
0 64 128 192
g
Case#9
h
Case#14
i
Objective Response Rate
PR SD PD
CAD
j
MKN45
EV CAD-WT CAD-D1371E
5-FU — + + +
p-H2A.X (S139)
DAPI
Merge
HCT116
EV CAD-WT CAD-D1371E
5-FU — + + +
p-H2A.X (S139)
DAPI
Merge
k
MKN45
EV CAD-WT CAD-D1371Y
5-FU — + + +
p-H2A.X (S139)
DAPI
Merge
HCT116
EV CAD-WT CAD-D1371Y
5-FU — + + +
p-H2A.X (S139)
DAPI
Merge

Supplementary Fig.5 Mutation information of CAD mutation samples detected by whole exon sequencing.

(**a**) Statistics on the number of variations in Case#9 and Case#14. 12471 mutations in Case#9 and 11524 mutations in Case#14. The median of the two samples was 11997.5.

(**b-c**) Box plot statistics for variation types and variance classification statistics of Samples. Through Maftools statistical analysis, it was found that a total of 9 mutations were found in the two samples (b), among which Missense-Mutation had a significantly higher number than other types, with a total of 21085 mutations, and Nonstop-Mutation had the least number (c).

(**d**) Variation type statistics. The number of Single Nucleotide Polymorphism (SNP) is significantly higher than other types, reaching a total of 20104. The Translocation Nucleotide Polymorphisms (TNP) type is the least, with 27 and 26 detected in Case#9 and #14, respectively.

(**e**) Statistics on SNPs. C>T had the highest number of mutations (42863), while T>A had the lowest number (3821).

(**f**) Statistics of the top ten gene sequences with mutations. Mutations in MUC17 and MUC6 gene sequences were significantly higher in both samples than in other genes, with a total of 176 and 135 mutations detected in both samples.

(**g-h**) IGV visualization highlighting CAD-D1371 mutant reads in WES data from chemotherapy-resistant GC samples (Case #9 and Case #14).

(**i**) Representative immunohistochemical images of CAD expression from clinical GC samples are shown. Scale bar, 50 μm.

(**j-k**) Representative Immunofluorescence of MKN45 and HCT116 cells expressing EV, CAD-WT, CAD-D1371E (j) or CAD-D1371Y (k), then treated with 5-FU. Scale bar, 20 μm.

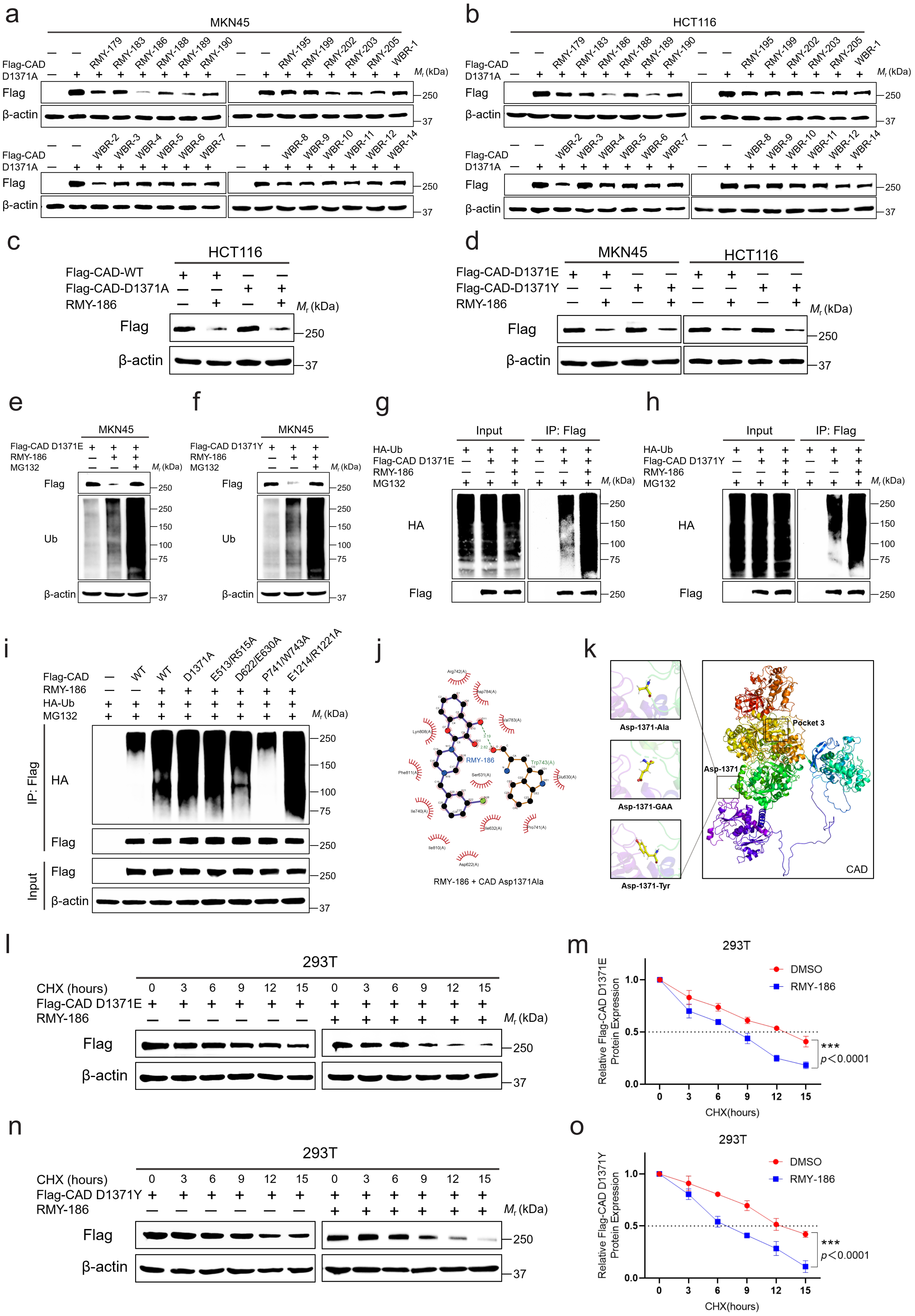

a
MKN45
Flag-CAD D1371A
RMY-179
RMY-183
RMY-186
RMY-188
RMY-189
RMY-190
RMY-195
RMY-199
RMY-202
RMY-203
RMY-205
WBR-1
WBR-2
WBR-3
WBR-4
WBR-5
WBR-6
WBR-7
WBR-8
WBR-9
WBR-10
WBR-11
WBR-12
WBR-14
Flag
β-actin
M_r (kDa)
250
37
b
HCT116
c
HCT116
Flag-CAD-WT
Flag-CAD-D1371A
RMY-186
d
MKN45
HCT116
Flag-CAD-D1371E
Flag-CAD-D1371Y
e
f
Flag-CAD D1371E
Flag-CAD D1371Y
MG132
Ub
150
100
75
g
h
Input
IP: Flag
HA-Ub
HA
i
Flag-CAD
WT
D1371A
E513/R515A
D622/E630A
P741/W743A
E1214/R1221A
j
RMY-186 + CAD Asp1371Ala
k
Asp-1371-Ala
Asp-1371-GAA
Asp-1371-Tyr
Asp-1371
Pocket 3
CAD
l
293T
CHX (hours)
m
DMSO
Relative Flag-CAD D1371E Protein Expression
CHX(hours)
***
$p < 0.0001$
n
o
Relative Flag-CAD D1371Y Protein Expression

Supplementary Fig.6 RMY-186 is a putative molecular inhibitor of CAD.

(**a-b**) Flag-CAD-D1371A was transfected into MKN45 (a) and HCT116 (b) cells and then treated with different small molecule compounds (10 μM) for 24 h. The expression levels of Flag-CAD-D1371A were determined.

(**c**) Flag-CAD-WT or Flag-CAD-D1371A was transfected into HCT116 cells and then treated with RMY-186 (10 μM) for 24 h.

(**d**) Flag-CAD-D1371E or Flag-CAD-D1371Y was transfected into MKN45 and HCT116 cells and then treated with RMY-186 (10 μM) for 24 h.

(**e-f**) Flag-CAD-D1371E (e) or Flag-CAD-D1371Y (f) overexpressed MKN45 cells were pre-treated with MG-132 (5 μM) then treated with RMY-186 (10 μM) for 24 h. Overexpressed protein of Flag-tag and ubiquitination were detected by immunoblotting.

(**g-h**) Immunoprecipitation showing ubiquitination of CAD-D1371E or CAD-D1371Y protein in HA-Ub and Flag-CAD-D1371E (g) or Flag-CAD-D1371Y (h) overexpressed MKN45 cells treated with RMY-186.

(**i**) Immunoprecipitation showing ubiquitination of CAD protein in MKN45 cells transfected with the indicated plasmids.

(**j**) The schematic illustration for the binding between CAD-D1371A and RMY-186 presented by LigPlot.

(**k**) Autodock predicts molecular docking of CAD mutation (AlphaFold2) with RMY-186.

(**l-o**) HEK-293T cells were transfected with Flag-CAD-D1371E (l-m) or Flag-CAD-D1371Y (n-o) and CHX (20 μg/ml) was added to cells 24 h after transfection with and

without RMY-186 (10 μM) treatment. Cells were collected after indicated time (0, 3, 6, 9, 12, or 15 h) and cell lystes were immunoblotted with anti-Flag antibody to detect the protein level change. Scanning densitometry was performed for each Western blot using ImageJ. The best-fit exponential decay lines were plotted using GraphPad Prism and the half-life can be read from it (marked by dot-dash line). n = 3 biologically independent samples per group.

Data in panels **e**, **f**, **g**, **h**, **i**, **l** and **n** derive from the same biological experiment but were processed on parallel gels (see Source Data for gel-specific details).

Data were expressed as means±SD, two-tailed Student's *t* test. ***p <0.001.

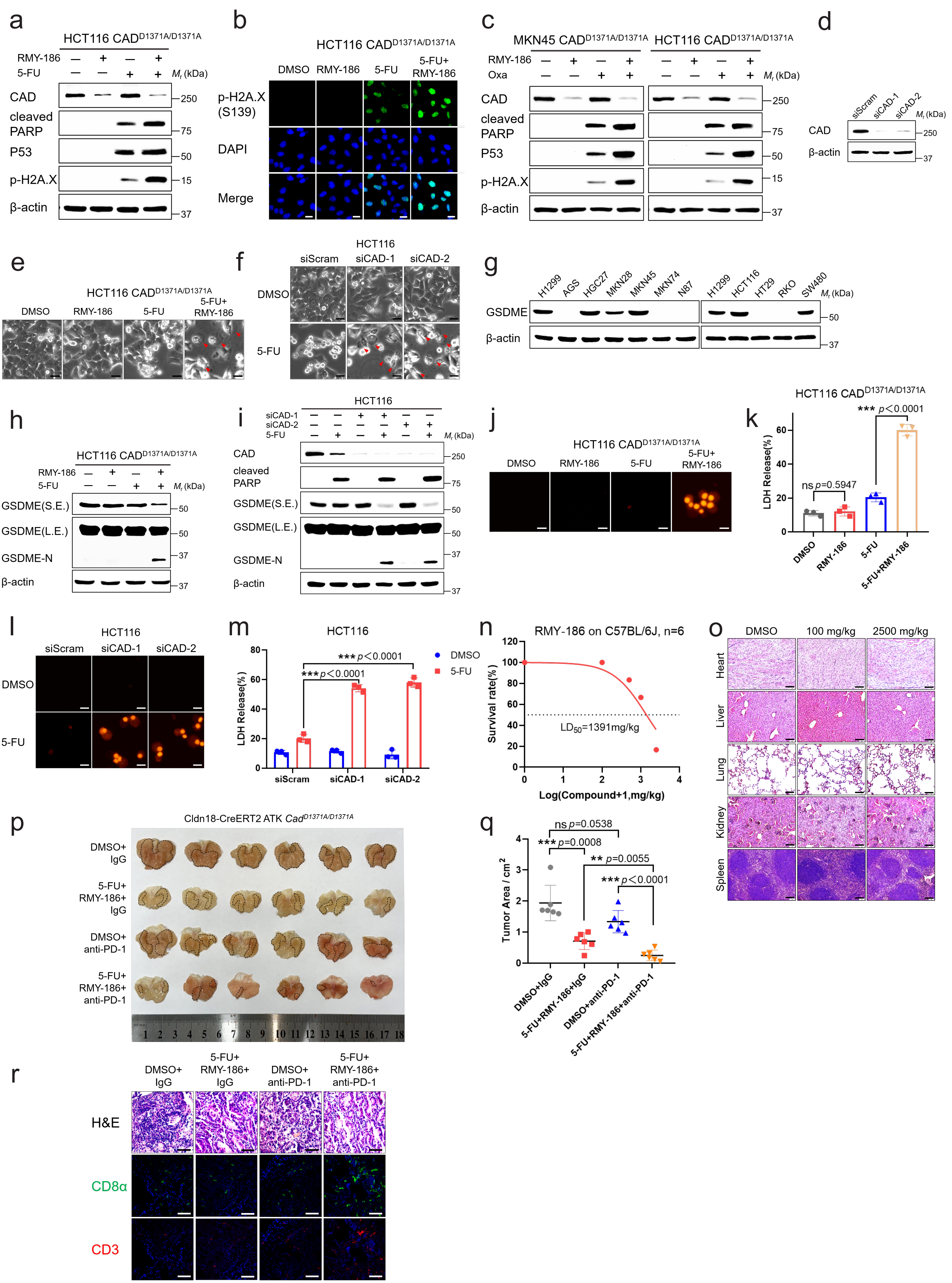
a
HCT116 CAD D1371A/D1371A
RMY-186
5-FU
Mr (kDa)
CAD
cleaved PARP
P53
p-H2A.X
β-actin
b
HCT116 CAD D1371A/D1371A
DMSO
RMY-186
5-FU
5-FU+ RMY-186
p-H2A.X (S139)
DAPI
Merge
c
MKN45 CAD D1371A/D1371A
HCT116 CAD D1371A/D1371A
RMY-186
Oxa
d
siScram
siCAD-1
siCAD-2
e
f
HCT116
siScram
siCAD-1
siCAD-2
g
GSDME
H1299
AGS
HGC27
MKN28
MKN45
MKN74
N87
HCT116
HT29
RKO
SW480
h
GSDME(S.E.)
GSDME(L.E.)
GSDME-N
i
siCAD-1
siCAD-2
j
k
LDH Release(%)
ns p=0.5947
*** p<0.0001
5-FU+RMY-186
l
m
DMSO
5-FU
*** p<0.0001
n
RMY-186 on C57BL/6J, n=6
Survival rate(%)
LD50=1391mg/kg
Log(Compound+1,mg/kg)
o
100 mg/kg
2500 mg/kg
Heart
Liver
Lung
Kidney
Spleen
p
Cldn18-CreERT2 ATK Cad D1371A/D1371A
DMSO+ IgG
5-FU+ RMY-186+ IgG
DMSO+ anti-PD-1
5-FU+ RMY-186+ anti-PD-1
q
Tumor Area / cm2
ns p=0.0538
*** p=0.0008
** p=0.0055
*** p<0.0001
DMSO+IgG
5-FU+RMY-186+IgG
DMSO+anti-PD-1
5-FU+RMY-186+anti-PD-1
r
H&E
CD8α
CD3

Supplementary Fig.7 RMY-186 eliminates resistance of chemo-insensitive cells.

(**a**) Protein expression of CAD, cleaved PARP, P53 and p-H2A.X was determined by Western blot after the HCT116 $CAD^{D1371A/D1371A}$ cells were treated with DMSO, RMY-186 (10 μM), 5-FU (50 μM), or the combination of RMY-186 and 5-FU for 24 h.

(**b**) p-H2A.X was determined by Immunofluorescence as described in (a). Scale bar, 20 μm.

(**c**) Protein expression of CAD, cleaved PARP, P53 and p-H2A.X was determined by Western blot after the MKN45 $CAD^{D1371A/D1371A}$ cells and HCT116 CAD^{D1371A} cells were treated with DMSO, RMY-186 (10 μM), Oxa (20 μM), or the combination of RMY-186 and Oxa for 24 h.

(**d**) HEK-293T cells were transfected with siCAD-1, siCAD-2, or universal negative control siRNA. Western blot was used to measure the levels of CAD.

(**e-f**) Microscopic images of pyroptotic cells. Arrowheads indicate ballooned cell membrane characteristic of pyroptotic cells. Scale bar, 25 μm.

(**g**) Protein expression of GSDME was determined by Western blot in multiple GC and CRC cell lines.

(**h**) Western blot was performed to detect the expression levels of GSDME in HCT116 $CAD^{D1371A/D1371A}$ cells with treatment as described in (a).

(**i**) Western blot was performed to detect the expression levels of GSDME in CAD-KD HCT116 cells with 5-FU treatment.

(**j**) PI (50 μg/ml) staining of pyroptotic cells in (a). Scale bar, 25 μm.

(**k**) Culture supernatants in (a) were collected to measure the percentage of LDH release.

n = 3 biologically independent samples per group.

(**l**) PI (50 μg/ml) staining of pyroptotic cells in (i). Scale bar, 25 μm.

(**m**) Culture supernatants in (i) were collected to measure the percentage of LDH release. n = 3 biologically independent samples per group.

(**n**) LD_{50} of RMY-186 tested on C57BL/6J mice, n=6 mice for each group, LD_{50} was estimated using non-linear fitting method.

(**o**) Small molecule compound RMY-186 displays favourable safety profile. H&E staining of tissues from mice treated with different concentrations of RMY-186 in an acute toxicity test, this experiment was performed 3 times independently with similar results. Scale bar, 100 μm.

(**p-q**) Whole-mount images (p) and the statistical analysis (q) of stomach from Cldn18-ATK $Cad^{D1371A/D1371A}$ mice in the indicated treatment groups. Tumors were marked with black dotted lines. n = 6 biologically independent samples per group.

(**r**) Tumor sections from (p) were subjected to immunofluorescence staining by anti-CD8α or anti-CD3 specific antibodies. Scale bar, 50 μm.

Data in panels **a**, **c**, **d**, **h** and **i** derive from the same biological experiment but were processed on parallel gels (see Source Data for gel-specific details).

Data were expressed as means±SD, two-tailed Student's *t* test. ns: not significant, $^{**}p$ <0.01, $^{***}p$ <0.001.

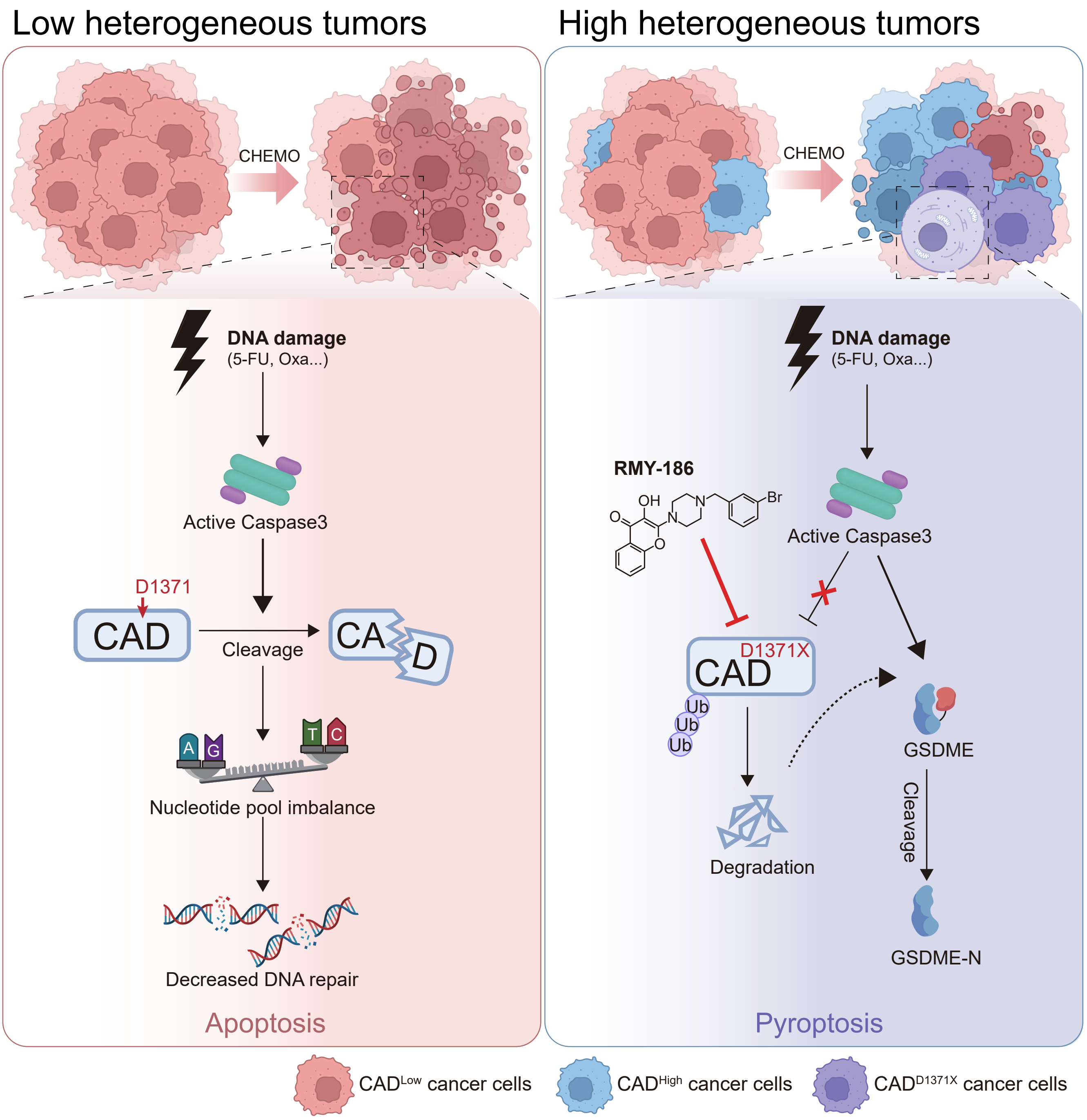

Low heterogeneous tumors
High heterogeneous tumors
CHEMO
CHEMO
DNA damage
(5-FU, Oxa...)
Active Caspase3
D1371
CAD
Cleavage
CA
D
A
G
T
C
Nucleotide pool imbalance
Decreased DNA repair
Apoptosis
DNA damage
(5-FU, Oxa...)
RMY-186
OH
O
N
N
Br
O
Active Caspase3
D1371X
CAD
Ub
Ub
Ub
Degradation
GSDME
Cleavage
GSDME-N
Pyroptosis
CAD^Low cancer cells
CAD^High cancer cells
CAD^D1371X cancer cells

Supplementary Fig. 8 Schematic of tumor heterogeneity affecting chemosensitivity. Endogenous apoptosis pathway activates downstream executive protein caspase-3 under the condition of chemotherapy-mediated DNA damage. CAD, the first rate-limiting enzyme of pyrimidine de novo synthesis pathway, is the physiological substrate of caspase-3. Activated caspase-3 cleaves CAD at Asp1371, which reduces the level of pyrimidine nucleotides. The imbalance of nucleotide pool intensifies DNA damage and finally initiates apoptosis. The tumor cells with high expression of CAD can resist apoptosis induced by chemotherapy to some extent by increasing pyrimidine metabolic flux. The high intrinsic heterogeneity of tumors and the reprogramming of pyrimidine metabolism led to the expansion of existing subclones and the emergence of new subclones during treatment, i.e., the expansion of tumor cells with high CAD expression and the emergence of tumor cells with CAD mutation at caspase-3 cleavage site. RMY-186 can bind to mutant CAD and promote its ubiquitination and degradation. On the basis of targeting CAD degradation, chemotherapy drugs can increase the cleavage of GSDME by activated caspase-3 to cause pyroptosis.

Supplementary Table 1. GC patient clinical characteristics and clinical response.

ID	Sex	Age at treatment start	Treated (Y/N)	CR, PR, SD, PD	TNM stage	Chemotherapy regimens
1	M	56	Y	PD	T4aN3aM0 pⅢB	FOLFOX
2	M	64	Y	PD	T4aN2M1 pIV	XELOX
3	M	58	Y	PD	T4aN3bM0 pⅢC	XELOX
4	M	56	Y	PD	T4aN3bM0 pⅢC	SOX
5	M	61	Y	PD	T3N3aM0 pⅢB	SOX
6	F	51	Y	PD	T4aN3bM0 pⅢC	SOX
7	F	36	Y	PD	T4aN3bM1 pIV	SOX
8	M	47	Y	PD	T3N1M0 pⅡB	SOX
9	F	62	Y	PD	/ cIVB	XELOX
10	M	67	Y	PD	/ cIVB	XP
11	F	48	Y	PD	/ cIVB	DX
12	M	63	Y	PD	/ cIVB	SOX
13	M	46	Y	PD	T4bN2M0 cIVA	SOX
14	M	61	Y	PD	T4aN0M0 ypⅡ	FOLFOX
15	M	55	Y	PD	T4bN3bM0 ypⅢ	XELOX
16	F	31	Y	SD	T4aN2M0 ypⅢ	SOX

Abbreviation: CR, complete response; PR, partial response; SD, stable disease; PD, progression of disease; TNM, tumor, node, and metastasis; FOLFOX, oxaliplatin, calcium folinate, and 5-fluorouracil; XELOX, oxaliplatin and capecitabine; SOX, oxaliplatin and tegafur; XP, capecitabine and cisplatin; DX, capecitabine and docetaxel.

Supplementary Table 2. Structures of small molecule compounds.

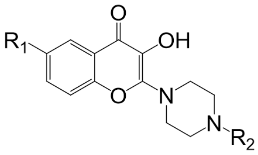

Compound	R1	R2	Compound	R1	R2	Compound	R1	R2
RMY-179	H	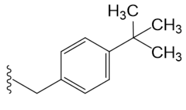	RMY-202	H	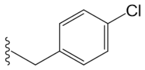	WBR-6	Br	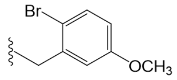
RMY-183	H	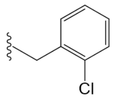	RMY-203	H	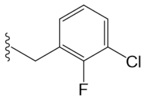	WBR-7	H	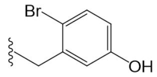
RMY-186	H	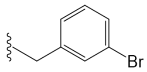	RMY-205	H	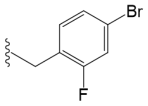	WBR-8	H	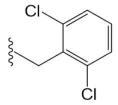
RMY-188	H	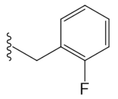	WBR-1	F	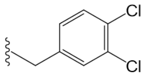	WBR-9	H	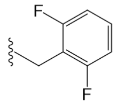
RMY-189	H	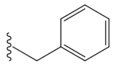	WBR-2	F	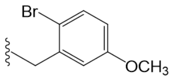	WBR-10	H	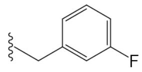
RMY-190	H	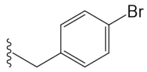	WBR-3	H	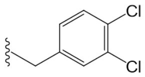	WBR-11	H	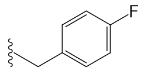
RMY-195	H	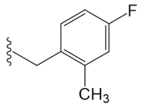	WBR-4	H	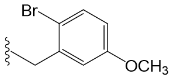	WBR-12	H	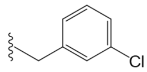
RMY-199	H	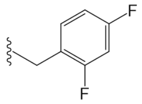	WBR-5	Br	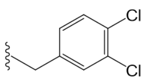	WBR-14	H	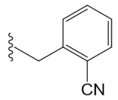
