# Supplementary material for: Cleavage of CAD by caspase-3 determines the cancer cell fate during chemotherapy
Source: Nat Commun. 2025 May 30;16:5006. doi: 10.1038/s41467-025-60144-2 (PMC12123037; doi:10.1038/s41467-025-60144-2)
Supplement: Supplementary file 4 — Reporting Summary [file 41467_2025_60144_MOESM4_ESM.pdf]

nature portfolio

Corresponding author(s): Funan Li, Yongxi Song, Zhenning Wang, Xuehui Hong

Last updated by author(s): May 8, 2025

Reporting Summary

Nature Portfolio wishes to improve the reproducibility of the work that we publish. This form provides structure for consistency and transparency in reporting. For further information on Nature Portfolio policies, see our Editorial Policies and the Editorial Policy Checklist.

Statistics

For all statistical analyses, confirm that the following items are present in the figure legend, table legend, main text, or Methods section.

n/a	Confirmed	
☐	☒	The exact sample size (*n*) for each experimental group/condition, given as a discrete number and unit of measurement
☐	☒	A statement on whether measurements were taken from distinct samples or whether the same sample was measured repeatedly
☐	☒	The statistical test(s) used AND whether they are one- or two-sided<br>*Only common tests should be described solely by name; describe more complex techniques in the Methods section.*
☒	☐	A description of all covariates tested
☐	☒	A description of any assumptions or corrections, such as tests of normality and adjustment for multiple comparisons
☐	☒	A full description of the statistical parameters including central tendency (e.g. means) or other basic estimates (e.g. regression coefficient) AND variation (e.g. standard deviation) or associated estimates of uncertainty (e.g. confidence intervals)
☐	☒	For null hypothesis testing, the test statistic (e.g. *F*, *t*, *r*) with confidence intervals, effect sizes, degrees of freedom and *P* value noted<br>*Give P values as exact values whenever suitable.*
☒	☐	For Bayesian analysis, information on the choice of priors and Markov chain Monte Carlo settings
☒	☐	For hierarchical and complex designs, identification of the appropriate level for tests and full reporting of outcomes
☒	☐	Estimates of effect sizes (e.g. Cohen's *d*, Pearson's *r*), indicating how they were calculated

Our web collection on statistics for biologists contains articles on many of the points above.

Software and code

Policy information about availability of computer code

Data collection	Real-time PCR system (Applied Biosystems), TimsTOF™ Pro Mass Spectrometer (Bruker, Germany), Flow cytometer (Fortessa X20; Becton, Dickinson and Company; US), Agilent 5400 system (AATI) (Agilent, USA), Monolith NT.115 (NanoTemper Technologies GmbH, Germany).
Data analysis	Graphpad Prism (version 8.0.1), Image J software program (version 1.38x), PyMOl Molecular Graphics system (Version 2.0 schrodinger, LLc.), the MassHunter software version B.07.01 SP1, GSNAP (version 2013-11-10), QX200 Droplet Microdroplet Analyzer, AlphaFold Protein Structure Database.

For manuscripts utilizing custom algorithms or software that are central to the research but not yet described in published literature, software must be made available to editors and reviewers. We strongly encourage code deposition in a community repository (e.g. GitHub). See the Nature Portfolio guidelines for submitting code & software for further information.

Data

Policy information about availability of data

All manuscripts must include a data availability statement. This statement should provide the following information, where applicable:
- Accession codes, unique identifiers, or web links for publicly available datasets
- A description of any restrictions on data availability
- For clinical datasets or third party data, please ensure that the statement adheres to our policy

Raw sequencing data for all samples have been uploaded to Sequence Read Archive (SRA) public database with accession number PRJNA1108914 (https://www.ncbi.nlm.nih.gov/bioproject/PRJNA1108914). RNA sequencing data are available under accession number SRX24491504 (https://www.ncbi.nlm.nih.gov/sra/

SRX24491504). The small-molecule crystallographic data for RMY-186 have been submitted to Cambridge Structural Database (CSD) with deposition number 2359201, and relevant information is deposited to PubChem (preview ID: 1553371761). The mass spectrometry proteomics data have been deposited to the ProteomeXchange Consortium via the iProX partner repository with the dataset identifier PXD059365 (https://proteomecentral.proteomexchange.org/cgi/GetDataset?ID=PXD059365). Source data are provided with this paper.

Research involving human participants, their data, or biological material

Policy information about studies with human participants or human data. See also policy information about sex, gender (identity/presentation), and sexual orientation and race, ethnicity and racism.

Reporting on sex and gender	119 patients with Gastric Cancer are involved in our study, which include 81 male and 38 female. The study received approval from the Institutional Ethics Review Board of the First Affiliated Hospital of China Medical University (Acceptance No. [2022] 366).
Reporting on race, ethnicity, or other socially relevant groupings	Tumor tissues and adjacent normal tissues from all Gastric Cancer patients (n=119) were collected at the First Affiliated Hospital of China Medical University between September 16, 2008, and May 22, 2022. Written informed consent was obtained from all donors, and each patient had undergone a pathology-based diagnosis of GC before surgery. Among these patients, samples from 16 chemoresistant patients were subjected to WES and ddPCR. Eight of these samples came from patients whose disease had progressed following adjuvant chemotherapy. The remaining eight samples were from patients clinically diagnosed with advanced GC, as indicated by CT staging, who had received neoadjuvant chemotherapy prior to surgery, but still showed disease progression. In these cases, samples were collected either through subsequent pathological biopsy or palliative surgery.
Population characteristics	see above
Recruitment	see above
Ethics oversight	The research protocol was approved by the Institutional Ethics Review Board of the First Affiliated Hospital of China Medical University (Acceptance No. [2022]366). The export of the relevant genetic information has been registered with and approved by China's Ministry of Science and Technology, with the First Affiliated Hospital of China Medical University serving as the authorized institution. All participating patients provided written informed consent, including consent for the publication of individual-level data.

Note that full information on the approval of the study protocol must also be provided in the manuscript.

Field-specific reporting

Please select the one below that is the best fit for your research. If you are not sure, read the appropriate sections before making your selection.

[x] Life sciences [ ] Behavioural & social sciences [ ] Ecological, evolutionary & environmental sciences

For a reference copy of the document with all sections, see nature.com/documents/nr-reporting-summary-flat.pdf

Life sciences study design

All studies must disclose on these points even when the disclosure is negative.

Sample size	No statistical methods were used to pre-determine sample sizes but the sample size was chosen in advance based on common practice of the described experiment in the literature and is specified for each experiment. For cell culture experiments, at least 3 replicates per group and for animal studies at least 8 animals per group were used unless specified otherwise, taking into account the variability within a cage and experimental group as well as between individual experimental repetitions to set appropriate sample numbers to allow for sound interpretation of experimental results.
Data exclusions	No data were excluded from our analyses.
Replication	Each experiment was conducted with biological and technical replicates and repeated at least three times for cell cultures studies. Trends were similar in all the replicates. All attempts at replication were successful. All data are representative of three independent experiments.
Randomization	Mice were randomly divided into different groups. No randomization was performed for other experiments as control group and treatment group in these experiments were defined.
Blinding	Data collection and analysis were not performed blind to the conditions of the experiments. Investigators were not blinded in cells, investigators performing experiments analyses was blinded to patients and animal genotyping information.

Reporting for specific materials, systems and methods

We require information from authors about some types of materials, experimental systems and methods used in many studies. Here, indicate whether each material, system or method listed is relevant to your study. If you are not sure if a list item applies to your research, read the appropriate section before selecting a response.

Materials & experimental systems

n/a	Involved in the study	
	☒	Antibodies
	☒	Eukaryotic cell lines
☒		Palaeontology and archaeology
	☒	Animals and other organisms
	☒	Clinical data
☒		Dual use research of concern
☒		Plants

Methods

n/a	Involved in the study	
☒		ChIP-seq
	☒	Flow cytometry
☒		MRI-based neuroimaging

Antibodies

Antibodies used

Antibodies against the following proteins were purchased from Cell Signaling Technology (Beverly, Massachusetts, USA): anti-CAD (#11933), anti-cleaved PARP (Asp214) (#9541), anti-caspase-3 (#9662), anti-caspase-6 (#9762), anti-caspase-7 (#9492), anti-LC3 I/II (#4108), anti-Phospho-Histone H2A.X (Ser139) (#9718), anti-FLAG (#8146), anti-HA (#3724), anti-β-actin (#3700), anti-DAPI (#4083). Antibodies against DHODH (#sc-166348), anti-UMPS (#sc-398086), anti-p53 (#sc-126) and anti-Ubiquitin (#sc-8017) were obtained from Santa Cruz Biotechnology (Dallas, Texas, USA). The antibody against CAD (#A6849) for immunohistochemistry was purchased ABclonal (Wuhan, China). The antibodies against GSDME (#ab215191), CD3 (#ab16669), and CD8 alpha (#ab 217344) were obtained from Abcam (Cambridge, UK). Antibodies against GOT1 (#14886-1-AP) and VDAC3 (#55260-1-AP) were obtained from Proteintech (Rosemont, USA). The dilution ratio for all of these antibodies was 1:1,000. Secondary antibodies used in this study were Goat Anti-Rabbit IgG H&L (#ab6721), Goat Anti-Mouse IgG H&L (#ab205719), and Rat Anti-mouse IgG for IP (HRP) (#ab131368, optimized secondary antibodies that do not detect denatured heavy and/or light chains during Western blot), which were obtained from Abcam. Secondary antibodies were used at a 1:5,000 dilution.

Custom rabbit anti-CAD-1-1371 (antigenic determinant: aa 358–KEATAGNPGGQTVR-371) and anti-CAD-△N1371 (antigenic determinant: aa 1831–TTTPERPRRGIPG-1843) polyclonal antibodies were custom synthesized against corresponding short peptides by GenScript (GenScript Biotech, Piscataway, NJ, USA).

Validation

All commercial antibodies were validated by manufactory. The validation information on their webpages are listed below:
Antibodies against the following proteins were purchased from Cell Signaling Technology (Beverly, Massachusetts, USA):
anti-CAD (#11933),
https://www.cellsignal.cn/products/primary-antibodies/cad-antibody/11933
anti-cleaved PARP (Asp214) (#9541),
https://www.cellsignal.cn/products/primary-antibodies/cleaved-parp-asp214-antibody/9541
anti-caspase-3 (#9662),
https://www.cellsignal.cn/products/primary-antibodies/caspase-3-antibody/9662
anti-caspase-6 (#9762),
https://www.cellsignal.cn/products/primary-antibodies/caspase-6-antibody/9762
anti-caspase-7 (#9492),
https://www.cellsignal.cn/products/primary-antibodies/caspase-7-antibody/9492
anti-LC3 I/II (#4108),
https://www.cellsignal.cn/products/primary-antibodies/lc3a-b-antibody/4108
anti-Phospho-Histone H2A.X (Ser139) (#9718),
https://www.cellsignal.cn/products/primary-antibodies/phospho-histone-h2a-x-ser139-20e3-rabbit-mab/9718
anti-FLAG (#8146),
https://www.cellsignal.cn/products/primary-antibodies/dykddddk-tag-9a3-mouse-mab-binds-to-same-epitope-as-sigma-aldrich-anti-flag-m2-antibody/8146
anti-HA (#3724),
https://www.cellsignal.cn/products/primary-antibodies/ha-tag-c29f4-rabbit-mab/3724
anti-β-actin (#3700),
https://www.cellsignal.cn/products/primary-antibodies/b-actin-8h10d10-mouse-mab/3700
anti-DAPI (#4083).
https://www.cellsignal.cn/products/buffers-dyes/dapi/4083

Antibodies against DHODH (#sc-166348),
https://www.scbt.com/zh/p/dhodh-antibody-e-8
anti-UMPS (#sc-398086),
https://www.scbt.com/zh/p/umps-antibody-a-9
anti-p53 (#sc-126),
https://www.scbt.com/zh/p/p53-antibody-do-1
anti-Ubiquitin (#sc-8017)
https://www.scbt.com/zh/p/ubiquitin-antibody-p4d1
were obtained from Santa Cruz Biotechnology (Dallas, Texas, USA).

The antibody against CAD (#A6849) for immunohistochemistry was purchased ABclonal (Wuhan, China).
https://abclonal.com.cn/catalog/A6849

The antibodies against GSDME (#ab215191),
https://www.abcam.cn/products/primary-antibodies/dfna5gsdme-antibody-epr19859-n-terminal-ab215191.html
CD3 (#ab16669, Abcam),
https://www.abcam.cn/products/primary-antibodies/cd3-epsilon-antibody-sp7-ab16669.html
CD8 alpha (#ab217344, Abcam),
https://www.abcam.cn/products/primary-antibodies/cd8-alpha-antibody-epr21769-ab217344.html
were obtained from Abcam (Cambridge, UK).

Antibodies against GOT1 (#14886-1-AP),
https://www.ptglab.com/products/GOT1-Antibody-14886-1-AP.html
VDAC3 (#55260-1-AP),
https://www.ptglab.com/products/VDAC3-Antibody-55260-1-AP.html
were obtained from Proteintech (Rosemont, USA).

Goat Anti-Rabbit IgG H&L (#ab6721),
https://www.abcam.cn/products/secondary-antibodies/goat-rabbit-igg-hl-hrp-ab6721.html
Goat Anti-Mouse IgG H&L (#ab205719),
https://www.abcam.cn/products/secondary-antibodies/goat-mouse-igg-hl-hrp-ab205719.html
Rat Anti-mouse IgG for IP (HRP) (#ab131368, optimized secondary antibodies that do not detect denatured heavy and/or light chains during Western blot),
https://www.abcam.cn/products/secondary-antibodies/mouse-igg-for-ip-hrp-ab131368.html
which were obtained from Abcam

Eukaryotic cell lines

Policy information about cell lines and Sex and Gender in Research

Cell line source(s)

All the cell lines employed in this research were purchased from the Shanghai Institute for Biological Science (China). HEK-293T cells were cultured in DMEM. GC cell lines (AGS, HGC27, MKN28, MKN45, MKN74 and NCI-N87), CRC cell lines (HCT116, HT29, RKO and SW480) and NSCLC cell line (NCI-H1299) were cultured in RPMI 1640.

Authentication

All cell lines used in this study were authenticated by short tandem repeat DNA finger printing.

Mycoplasma contamination

All cell lines used in this study were routinely tested for mycoplasma contamination and all tested negative for mycoplasma.

Commonly misidentified lines
(See ICLAC register)

No commonly misidentified cell lines were used in this study.

Animals and other research organisms

Policy information about studies involving animals; ARRIVE guidelines recommended for reporting animal research, and Sex and Gender in Research

Laboratory animals

Cldn18-CreERT2; Apcfl/fl; Trp53fl/fl; KrasG12D (Cldn18-ATK) mice were a generous gift from Dr. Yoshiaki Ito (National University of Singapore), and the mice developed obvious hyperplastic gastric tumors after Tamoxifen induction. caspase-3 KO mice were a generous gift from Dr. Jianfeng Wu (Xiamen University). CadD1371A/D1371A mice were constructed by the Xiamen University Laboratory Animal Center. Eight weeks old Cldn18-ATK mice, Cldn18-ATK caspase-3 KO mice and Cldn18-ATK CadD1371A/D1371A mice were induced with a single intraperitoneal injection of 100 mg/kg tamoxifen (#T5648, Sigma) dissolved in corn oil (#HY-Y1888, MedChemExpress). Two weeks after Tamoxifen induction, 5-FU (#F6627, Sigma) was injected intraperitoneally at a dosage of 25 mg/kg once weekly for a month.
For RMY-186 treatment, eight weeks old C57BL/6 mice were used to evaluate the acute toxicity (LD50) of RMY-186 in the living body. 100, 250, 500, 1000, and 2500mg/kg of RMY-186 were dissolved in normal saline containing 5% DMSO and 1% carboxymethylcellulose. These mice were randomly divided into six groups and received vehicle control or different concentrations of RMY-186 (six mice in each group). The test substance was given through intraperitoneal injection, and mortality over 24 hours was observed. RMY-186 at a concentration of 100mg/kg is administered to Cldn18-ATK CadD1371A/D1371A mice, whether or not it is combined with 5-FU.
To evaluate the therapeutic effects of combination treatment with RMY-186 and anti-PD-1 antibodies under chemotherapy. Cldn18-ATK mice were randomly assigned into four groups (six mice in each group) after Tamoxifen induction. Mice were administered with 5-FU and RMY-186 in the presence or absence of IgG2a (#BE0085, Bio X Cell, AB_1107771) or anti-PD-1 antibodies(CD279) (#BE0146, Bio X Cell, AB_10949053). Anti-PD-1 antibodies (200 μg/mouse) were injected intraperitoneally twice a week for a month.

Wild animals

There were no wild animals used.

Reporting on sex

This study did not involve reporting on sex.

Field-collected samples

This study did not involve field-collected samples.

Ethics oversight

All the animal experiments below were approved by the Animal Ethics Committee of Xiamen University (Acceptance No. XMULAC20200080). The ethics protocol explicitly defined humane endpoints, stipulating a maximal permissible tumour burden not exceeding 20 mm in diameter or strictly limiting maximal tumour volume to ≤10% of the animal's body weight to ensure animal welfare. Animals were housed under a standardized 12-hour light/12-hour dark cycle and provided with ad libitum access to food and

water throughout the experimental period.

Note that full information on the approval of the study protocol must also be provided in the manuscript.

Clinical data

Policy information about clinical studies

All manuscripts should comply with the ICMJEguidelines for publication of clinical research and a completedCONSORT checklist must be included with all submissions.

Clinical trial registration	N/A
Study protocol	N/A
Data collection	N/A
Outcomes	N/A

Plants

Seed stocks	N/A
Novel plant genotypes	N/A
Authentication	N/A

Flow Cytometry

Plots

Confirm that:

- [x] The axis labels state the marker and fluorochrome used (e.g. CD4-FITC).
- [x] The axis scales are clearly visible. Include numbers along axes only for bottom left plot of group (a 'group' is an analysis of identical markers).
- [x] All plots are contour plots with outliers or pseudocolor plots.
- [x] A numerical value for number of cells or percentage (with statistics) is provided.

Methodology

Sample preparation	Cell cycle distribution was documented employing a flow cytometer (Fortessa X20; Becton, Dickinson and Company; US) with a Cell Cycle and Apoptosis Analysis Kit (#40301ES; Yeasen) according to the instructions of the manufacturer. The cells were then trypsinized with trypsing-EDTA and then fixed employing 70% alcohol for 24 hours at 4 °C. Thereafter, the cells were washed once or twice with 1×PBS, freshly prepared, and then resuspended in the staining solution and then combined with the PI and RNase A solution mixture. The specimens were placed at 37 °C in the darkness with a duration of 30 minutes, and then flow cytometry was performed.
Instrument	Flow cytometer (Fortessa X20; Becton, Dickinson and Company; US)
Software	FlowJo vX10
Cell population abundance	A total of 10,000 single cells were analyzed per sample.
Gating strategy	Cells were identified with FSC-A/SSC-A gating and followed by FSC-A/FSC-H for singlets. An identical cell gating strategy was applied to all samples analyzed at the same time. A figure exemplifving the gating strategy would be provided upon reguest.

- [x] Tick this box to confirm that a figure exemplifying the gating strategy is provided in the Supplementary Information.
